# Supplementary material for: End Sequence Analysis Toolkit (ESAT) expands the extractable information from single-cell RNA-seq data
Source: Genome Res. 2016 Oct;26(10):1397–410. doi: 10.1101/gr.207902.116 (PMC5052061; doi:10.1101/gr.207902.116)
Supplement: Supplemental Material [file supp_gr.207902.116_Supp_Materials.tar.gz › ESAT-esat_v0.1/doc/html/functions_vars_u.html]

ESAT: Class Members - Variables


ESAT


- Main Page
- Related Pages
- Packages
- Classes
- Files

- Class List
- Class Index
- Class Hierarchy
- Class Members

- All
- Functions
- Variables
- Enumerator

- a
- b
- c
- d
- e
- f
- g
- h
- i
- k
- l
- m
- n
- o
- p
- q
- r
- s
- t
- u
- v
- w


All Classes Namespaces Files Functions Variables Enumerator Pages

### - u -

- UNGAPPED\_ALPHABET\_SIZE
  : broad.core.multiplealignment.MultipleAlignment
- UNKNOWN
  : umms.core.annotation.Annotation.Strand
- UP
  : jaligner.Directions
- USAGE
  : broad.core.annotation.AnnotationUtils
  , broad.core.math.EmpiricalDistribution
  , broad.core.math.Statistics
  , broad.core.motif.PositionWeightMatrixIO
  , broad.core.motif.PWMUtils
  , broad.core.motif.SequenceMotif
  , broad.core.motif.SequenceMotifCluster
  , broad.core.multiplealignment.AlignmentExtractor
  , broad.core.multiplealignment.MAFAlignment
  , broad.core.sequence.Extractor
  , broad.core.siphy.PiHMM
  , broad.core.siphy.TreeScaler
  , broad.pda.samtools.FilterFastqReads
  , broad.pda.samtools.PairedEndTDF
  , broad.pda.samtools.TrimFastqReads
  , broad.pda.seq.rap.BuildRatioNullDistribution
  , broad.pda.seq.rap.CollectAnnotationEnrichments
  , broad.pda.seq.rap.CountReads
  , broad.pda.seq.rap.GCContent
  , broad.pda.seq.rap.PlotAggregateRegions
  , broad.pda.seq.rap.RatioPermutationPeakCaller
  , broad.pda.seq.rap.rna.CollectRnaSeqMetrics
  , broad.pda.seq.rap.rna.CountReads
  , broad.pda.seq.rap.ShuffleBAM
  , broad.pda.seq.rap.ShuffleBED
  , broad.pda.seq.rap.SkellamKLPeakCaller
  , broad.pda.seq.rap.SlideAndCalculate
  , broad.pda.seq.rap.SlideAndCount
  , broad.pda.seq.rap.UnmarkDuplicates
- USE\_INTERMEDIATES
  : broad.pda.seq.rap.BuildRatioNullDistribution


---

Generated on Fri Aug 15 2014 15:11:17 for ESAT by  

 1.8.7
